# On the Treatment of Fevers and Inflammations without Mercury

**Published:** 1847-02

**Authors:** A. G. Henry

**Affiliations:** Pekin, Illinois


ILLINOIS AND INDIANA

MEDICAL AND SURGICAL JOURNAL.

Vol. I. NEW SERIES.—FEBRUARY, 1846. No. 6.

### PART I.—ORIGINAL COMMUNICATIONS.

### ARTICLE I.

*On the Treatment of Fevers and Inflammations without Mercury.* By A. G. Henry, M.D., of Pekin, Illinois.

It is no intention of mine to join in the crusade (now going on in the ranks of the advocates of the botanical system,) against mercury, as a remedial agent in the treatment of disease; but my object is, to show that it can be dispensed with in the treatment of those forms of disease, in which it has been regarded by the great mass of the profession as indispensable; and if possible, contribute something toward correcting the abuse of one of the most valuable remedies in our materials of medicine. The indiscriminate use of calomel by the profession in the Valley of the Mississippi for years past, has rendered them as obnoxious to the charge of quackery, as the Thompsonian, who prescribes his lobelia in every form of disease. That great mischief has resulted to the community from its abuse, cannot be questioned; and if we have a remedy of greater power, that does not produce any of those unpleasant consequences which so often follow the mercurial plan of treatment, it will no doubt be acceptable to the community, and readily received by the profession.

The experience of the last ten years has satisfied me, that we have, in four and six grains of opium, a remedy of far greater power in the treatment of fevers and inflammations, than mercury; and which can be substituted for it with perfect safety, and save to our patients an immense amount of suffering.

I claim to have been the first to call the attention of the profession to the use of from *four to six* grains of opium in fevers and inflammatory affections; and I have for the last ten years insisted most strenuously in my intercourse with medical men, that the evil effects, or want of benefit resulting from the use of opium in acute forms of disease, was attributable to the fact of its being given in too small doses. I am happy to find myself sustained in this opinion by Dr. Griffin, in a recent publication on the use of opium in abdominal affections, where he ventured upon its use to the extent of three grains of opium every two hours, with the most happy results. I have for many years administered it in doses of from four to six grains in cases of inflammatory dysentery after bleeding, and repeating the dose in three or four hours, if the first did not entirely subdue the disease, *which it will do* in nine cases out of ten.

When the practice receives the partial sanction of such names of Stokes, Bell, and Griffin, I shall expect that the suggestions of an obscure country practitioner will secure from the profession a sufficient degree of consideration to induce a trial of the remedy.

In an article upon the use of *large doses* of opium in fevers and dysentery, published in the August No. of the Missouri Medical and Surgical Journal, 1845, I submitted the following propositions as the results of long observation and experience in the use of large doses of opium; and their correctness has been confirmed by subsequent experience.

1st. That there is more danger of injurious consequences resulting from the use of one or two grains of opium in fever and inflammation, than from four to six grain doses.

2d. That two and three grain doses will often aggravate the symptoms and do positive injury, when a four or six grain dose would secure a favorable crisis in the disease.

3d. That *ten* grains of calomel, combined with four or five grains of opium, will act more decidedly and promptly on the secretory system, than thirty grains without the opium, or with one or two grains; assuming that both shall remain in the system the same length of time without passing off by the bowels.

4th. That a five grain dose will not constipate the bowels as much as a one grain dose, *neither will it produce as much sleep* as a grain and a half, or two grains.

5th. That when you produce ptyalism by combining large doses of opium with your calomel, you produce it more promptly, and avoid all danger of profuse salivation, with mercurial irritation.

Up to the time of the publication of that article, I had most generally combined calomel with my opium for the purpose of securing a cathartic effect after the influence of the opium had passed off, but not with a view of producing ptyalism. But having about that time removed from Springfield to a neighborhood where calomel was used by the faculty as the main remedy in every form of acute disease; and where its injurious effects were plainly visible in the broken down constitutions of very many of the adult population, induced by often repeated salivations, I was led in such cases to forego its use entirely, and the result has been most satisfactory.

When called to treat an ordinary case of bilious remittent fever, if there is high arterial action, I use the lancet, followed by an emetic of ipecacuanha and tartar, given so as to produce an emetico-cathartic effect; and, as soon as the stomach will retain it, give a four or six grain dose of opium, and repeat it in the course of three or four hours, if the first does not produce full diaphoresis. The next morning I give the black draught, or salts and cream tartar, until the bowels are freely moved, and at night repeat the dose of opium. The patient rests quietly during the night, and I confidently expect in the morning to find a full intermission; when one or two five grain doses of quinine puts an end to the fever, and secures a rapid convalesence, free from all those distressing consequences which are so apt to follow the mercurial plan of treatment.

The results of this practice are most satisfactory in fevers with gastric irritation, or abdominal affections, which are found so difficult of management, and which so often prove fatal, when treated on the most approved plan hitherto recommended to the profession.

In such cases, a full bleeding from the arm, with cups to the epigastrium, followed by a four or five grain dose of opium during the first twenty-four hours of the attack, seldom fails to secure permanent relief.

My object in this communication is to show that fevers and inflammations can be safely and successfully treated by substituting large doses of opium for calomel, now so generally relied upon by the profession, particularly in fever. But where it can be used without danger of salivation, I would not object to ten or twelve grains being given with the opium, in the course of the twenty-four hours; for it must be conceded that we have no better cathartic in febrile affections than calomel. Still in those cases where I have dispensed with its use entirely, I have secured apparently as favorable results.

It is the relying upon it as the *main remedy* to which I most strenuously object, and not to its being used with proper discrimination.

Since 1836, when I first ventured upon the use of five grain doses of opium, I have relied upon them with great confidence in the treatment of all acute pneumonic affections. After a full bleeding at the commencement of the attack, I give nauseating doses of tartar until the bowels are feely acted upon, when I administer a four or six grain dose of opium, and repeat it, if necessary, in the course of four or six hours. Saline laxations, with expectorants, will generally complete the cure without a repetition of the bleeding, or opiate.

I have often subdued the most violent attacks of pleuritis, with a single bleeding, followed by a full dose of opium.

The suggestions of Professor Barbour, of giving the opiate a short time previous to the bleeding, I am inclined to think is an improvement upon my practice, in pneumonic inflammations; but I cannot speak confidently from experience.

I am satisfied that the antiphlogistic power of opium has never yet been properly appreciated by the profession; but I find it now beginning to attract the attention of the faculty. venture the prediction, that in a few years more, it will be regarded by the profession as a remedial agent of far more power, when given in full doses, in the treatment of fevers and inflammations, than mercury.

As a remedy in enteritis and peritonitis, it is invaluable; and although not generally admissible in inflammations of the cerebro-spinal system, still, in those cases, it can be used with safety and advantage simultaneously with blood-letting, in the onset of the attack. In all cases of cerebral affections occurring in the latter stages of fevers, resulting from loss of power, a *full dose* will change the entire complexion of the case in a few hours, and place the patient in a state of safety.

For a more full and detailed history of my mode of using opium in fever, and particularly those of a typhoid and congestive character, I must refer the profession to my article published in the Missouri Medical Journal for August, 1845.

I am fully aware of the deep rooted prejudices that exist in the minds of medical men against the use of opium, and especially so against the *dose* which I recommend. But I know them to be entirely groundless. I have in the course of the last ten years administered four and six grain doses to thousands of patients, under almost every variety of circumstances, without in any one instance experiencing any unpleasant consequences from an over dose. I am fully convinced from observation, that cases do occur where a four or six grain dose could be given once or twice in the twenty-four hours with safety, where two grain doses, repeated every three or four hours, until ten or twelve grains had been given, would prove fatal.

I have purposely avoided in this as well as in my former article, entering into an examination of the "modus operandi" of five grain doses of opium, preferring to leave that question to be settled by each member of the faculty, as may best suit his preconceived opinions; believing that *facts* are now more eagerly sought for by the profession, than theories.

Should any gentleman, however, object to giving the practice a trial, for want of a *philosophical theory* to sustain it, and will make his objections known through the columns of your Journal, I will do my utmost to supply the deficiency.

*Pekin, Ill. November 26th,* 1846.

